# ABA and Melatonin: Players on the Same Field?

**DOI:** 10.3390/ijms252212266

**Published:** 2024-11-15

**Authors:** Ivan Bychkov, Natalia Kudryakova, Elena S. Pojidaeva, Anastasia Doroshenko, Victoria Shitikova, Victor Kusnetsov

**Affiliations:** K.A. Timiryazev Institute of Plant Physiology RAS, 35 Botanicheskaya St., Moscow 127276, Russia; ivan.a.b@mail.ru (I.B.); alenapoj@mail.ru (E.S.P.); anastasiya04101993@gmail.com (A.D.); vicry@yandex.ru (V.S.); vkusnetsov2001@mail.ru (V.K.)

**Keywords:** *Arabidopsis thaliana*, abscisic acid, gene expression, high light stress, melatonin, mutants

## Abstract

In plants, abscisic acid (ABA) and melatonin (MT) are conventionally treated as molecules mitigating stress responses. To understand the mechanisms of ABA–MT interplay, we examined the effects of ABA and MT treatment in ABA and MT loss-of-function mutants of *Arabidopsis thaliana* exposed to high light (HL) stress. ABA constantly suppressed *ASMT* encoding N-acetylserotonin methyltransferase in the context of differential responses of other MT biosynthesis genes in both the wild type (WT) and mutants. However, this response was absent in the mutant with the disrupted ABI4. Given that the *ASMT* promoter region contains several potential ABI4-binding elements, these data suggest that *ASMT* can be a potential target gene for ABI4. A role for ABI4 in the interactions between ABA and MT is supported by the finding that *ABI4* is constitutively derepressed in the MT signaling mutants *cand2* and *gpa1*, which exhibited elevated steady state levels of *ABI4* transcripts and were not regulated by either stress or melatonin. In addition, the *abi4* mutant showed increased modulations in the expression of the MT catabolic genes *M2H* and *M3H* in response to ABA treatment, inferring that this transcription factor is a negative regulator of ABA-dependent changes in MT content. Furthermore, all tested mutants with impaired ABA synthesis or signaling displayed elevated steady state MT levels compared to WT, while MT treatment contributed to the downregulation of key ABA synthesis and signaling genes. Collectively, our results suggest that ABA and melatonin act antagonistically, modulating the expression of ABA and MT signaling and metabolism genes. To understand the mechanisms of ABA–MT interactions, we studied the effects of ABA and MT treatment in ABA and MT loss-of-function mutants of *Arabidopsis thaliana* exposed to severe light stress (SLS).

## 1. Introduction

Melatonin is a multifunctional effector that plays diverse physiological roles in plants, from seed germination to post-harvest fruit storage and seed longevity [1]. However, the beneficial effects of this molecule as an anti-stress agent are of paramount importance due to its powerful antioxidant activity. Melatonin acts as a scavenger of reactive oxygen species (ROS) and reactive nitrogen species (RNS), such as O_2_^−^, OH^−^, NO, and ONOO^−^, and also enhances the expression of superoxide-generating enzymes (RbOHs), superoxide dismutase, and the enzymes involved in the detoxification of excess H_2_O_2_, particularly CAT, POD, and APX [2]. At low concentrations, melatonin may act as a hormone-like signaling molecule involved in crosstalk with virtually all known plant hormones by modulating their signaling circuits and metabolism [3].

The *Arabidopsis* genome harbors several genes encoding four successive steps of melatonin biosynthesis. During the first two steps, serotonin is derived from tryptophan by two enzymatic reactions carried out by TDC (tryptophan decarboxylase) and T5H (tryptamine 5-hydroxylase). The next step is committed SNAT (serotonin N-acyltransferase) responsible for the synthesis of *N*-acetylserotonin, which is further catalyzed into melatonin by *O*-methyltransferases COMT (caffeic acid O-methyltransferase) and ASMT (N-aceylserotonin O-methyltransferase) [4]. Two melatonin metabolic genes *M2H* (melatonin 2-hydroxylase) and *M3H* (melatonin 3-hydroxylase) convert melatonin into 2-hydroxymelatonin and cyclic 3-hydroxymelatonin, respectively [5].

The signaling pathway of melatonin and its metabolites is proposed to act through the mitogen-activated protein kinase (MAPK) cascade, which is preceded by receptor-like kinases (RLKs), putative candidates for melatonin receptors in plants [6]. An alternative signaling circuit includes the putative MT receptor gene *CAND2* (also known as *PMTR1*), which is associated with *GPA,* encoding the α-subunit of the heterotrimeric G protein [7].

Abscisic acid is conventionally considered a stress hormone since its level is greatly increased under a variety of abiotic stresses [8]. ABA via binding to the PYR/PYL/RCAR (PYL) family of receptors, inhibits a class of A-type protein phosphatase 2Cs (PP2Cs). PP2C inactivation triggers the phosphorylation of a collection of basic leucine zipper transcription factors (ABA-responsive element binding factors, ABFs) by a small set of class 3 sucrose nonfermenting-1-related protein kinase 2s (SNRK2s). Phosphorylated ABFs activate downstream target genes through the *cis*-acting ABA response elements (ABREs). In addition to the canonic PYL-PP2C-SnRK2 core pathway, ABA responses in plants require a network of other signaling pathways that involve calcium, ROS, NO, phospholipids, and the various kinases [9]. All these pathways can interact with each other through positive or negative feedback mechanisms producing a remarkable pleiotropy of effects in plant responses to abiotic stresses.

Melatonin-mediated action generally induces the downregulation of ABA biosynthesis genes and the upregulation of ABA catabolism genes, resulting in a decrease in ABA levels [2,3]. For example, in apple melatonin selectively down-regulated *MdNCED3*, an ABA synthesis gene and triggered its catabolic genes, *MdCYP707A1* and *MdCYP707A2*, reducing ABA contents in drought-stressed plants [10]. Under drought stress, exogeneous melatonin downregulated the *NCED1* expression in maize and induced the expression of *ABA8ox1 (ABA-8- oxidase 1)* and *ABA8ox3* responsible for the ABA breakdown [11]. Other examples include Chinese cabbage [12], Chinese hickory *Carya cathayensis* [13], mango [14], cucumber [15], and ryegrass [16]. A special mechanism by which melatonin antagonizes ABA action was recently described in *Arabidopsis*. Melatonin offsets ABA action to delay leaf senescence via RBOHD-dependent H_2_O_2_ production that triggers [Ca^2+^]cyt accumulation and subsequently inhibits K^+^ efflux and delays cell death/leaf senescence in response to ABA [17].

On the other hand, an increase in melatonin content can lead to the upregulation of ABA-responsive genes testifying that crosstalk between MT and ABA is highly variable. Opposite responses were detected for *Elymus nutans* [18], *Brassica napus* [19], and *Raphanus sativus* [20]. The overexpression of *AtASMT* caused massive melatonin accumulation and synergized with ABA to inhibit seed germination in *Arabidopsis* [21]. In the case of barley, exogenously applied melatonin resulted in a higher ABA concentration in the drought-primed plants than in the nonprimed plants when exposed to cold stress [22]. Likewise, during arsenic stress, MT treatment of the susceptible rice cultivar Khitish up-regulated transcript levels of *NCED3*, and down-regulated transcript levels of *ABA8ox1*, elevating the endogenous ABA level. However, the ABA concentration remained unaltered in the tolerant rice cultivar Muktashri, indicating that the response depends upon the plant genotype [23].

While much of the work conducted on melatonin–ABA interplay in plants has been targeted on the influence of melatonin on ABA content and signal transduction, few studies have focused on the intersecting effects of ABA and melatonin on the expression of signaling and metabolic genes of these regulators. In rice, melatonin was shown to regulate ROS homeostasis under nitrogen limitation via the module OsbZIP79-OsABI5, composed of two ABA-related *trans*-factors. However, the module did not directly regulate melatonin biosynthesis [24]. On the other hand, treatments with abscisic and methyl jasmonic acids simultaneously induced three isoforms of N-acetylserotonin methyltransferase in rice, the final enzyme in melatonin biosynthetic pathway [25].

Although light is essential for the survival of plants, the exposure of a plant to excessive light leads to the inactivation of photosynthetic functions and the production of ROS and, as a consequence, affects organelle and nuclear gene expression. To reduce photoinhibition and the production of ROS, the plant has evolved an efficient scavenging system composed of enzymatic and nonenzymatic scavengers. Additional HL-specific responses comprise the adjusting of light-harvesting antennae size and the dissipation of excessively absorbed light by non-photochemical quenching as well as the expression of early light-induced proteins (ELIPs), which may bind chlorophyll *a* and lutein [26]. Acclimation to HL also depends on the transformation of retrograde signals from the chloroplasts, which in turn, depend on hormonal signals including abscisic acid, salicylic acid, and the precursor of jasmonic acid, 12-oxophytodienoic acid [27]. It should also be noted that, although HL stress has its intrinsic manifestations, stress-sensing pathways induced by various stresses may converge due to the existence of cross tolerance in plants. Therefore, within certain limitations, the mechanisms and pathways that regulate and coordinate plant responses to HL stress can be extrapolated to other types of abiotic stresses.

In this work, using exogenously applied ABA and melatonin as well as melatonin and ABA-related mutants, we showed that ABA is involved in the downregulation of the *Arabidopsis ASMT* gene under high light stress via the activity of the transcription factor ABI4 (ABSCISIC ACID INSENSITIVE 4). Additionally, we identified that the MT-dependent regulation of ABA genes depends on the *CAND2*-*GPA1* signaling circuit.

## 2. Results

### 2.1. ABA Treatment Differentially Regulates Physiological Parameters and Stress-Induced Genes in Col0 and MT Mutants 

**Figure 1:**
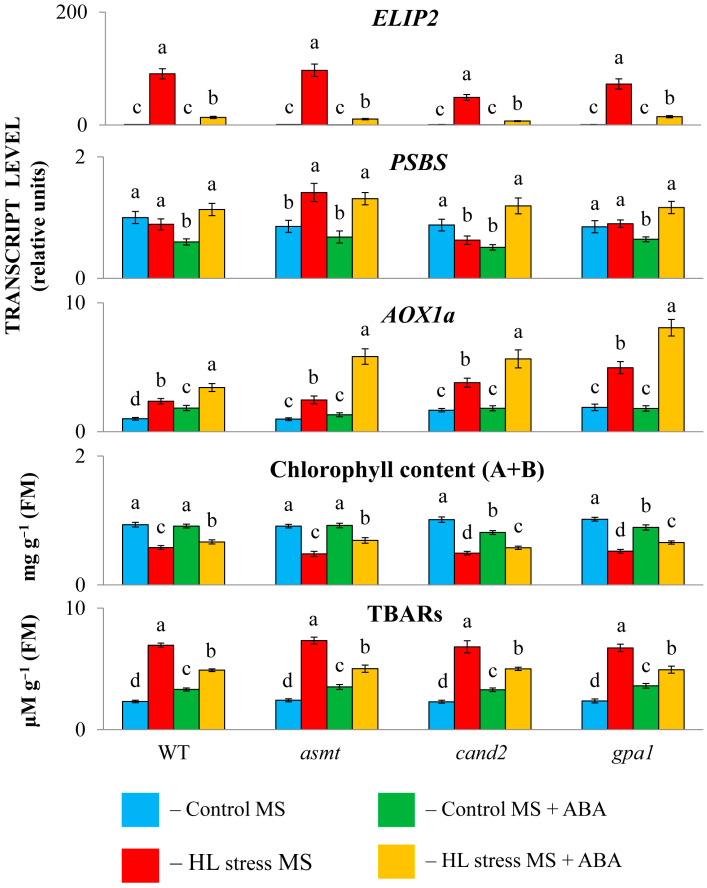
The effect of high light stress and ABA treatment on the TBAR content, chlorophyll content, and the expression of stress marker genes. WT and mutant plants were grown on a Murashige and Skoog (MS) medium in Petri dishes for two weeks under a 16 h light/8 h dark photoperiod at 23 °C with 60 μmol m^−2^ s^−1^ (control). ABA was used for treatment at a concentration of 50 μM. Experimental plants were exposed to high light (HL) for 24 h at 600 μmol m^−2^ s^−1^ (stress). The data presented in the figure are the mean values (*n* ≥ 3). Material was extracted and pooled from 7 to 10 plants for each of the biological replicate. Error bars represent SEs. Different letters denote statistically significant differences among variants within the same genotype at *p* < 0.05 (ANOVA with post hoc Tukey’s multiple-comparison test).

ABA treatment under mild light (60 μmol m^−2^ s^−1^, 24 h) was perceived by wild type plants as a mild stress, as evidenced by the reduced transcript accumulation of *PSBS* involved in quenching singlet-excited chlorophylls and the increased expression of the stress marker gene *AOX1a*, which participates in the fine-tuning of mitochondrial membrane potential and the alleviation of ROS production (Figure 1). WT plants exposed to HL stress (600 μmol m^−2^ s^−1^, 24 h) experienced severe damage, as can be seen in Figure S1. Under HL stress, ABA additionally increased the accumulation of *AOX1a* transcripts and also reduced stress-induced levels of *ELIP2* transcripts (EARLY LIGHT-INDUCED PROTEIN 2), which is known to modulate chlorophyll synthesis to prevent photo-oxidative stress [28]. MT mutants, when treated with ABA, did not differ significantly from the wild type in their *PSBS* and *AOX1a* responses under either control or stress conditions.

In parallel, the content of TBARs, a marker of membrane damage caused by lipid peroxidation, was significantly lifted by the ABA treatment of plants under mild light, whereas it was decreased under the combined application of HL and ABA compared to stress-induced levels. Hence, the ABA treatment of control plants had a positive effect only under stress conditions. Exogenous ABA did not significantly change chlorophyll content in WTs and *ASMT* mutants under mild light but promoted increased chlorophyll levels under excessive light compared to stress-repressed levels. On the other hand, MT signaling mutants, *cand2* and *gpa1*, exhibited reduced chlorophyll levels under mild light when treated with ABA, which indicates their elevated sensitivity to the hormone. However, as with the wild type, the chlorophyll content of these mutants increased after ABA treatment compared to their HL control values, confirming the plasticity of plant responses to ABA.

### 2.2. Responses of MT-Related Genes to ABA Treatment of MT Mutants

**Figure 2:**
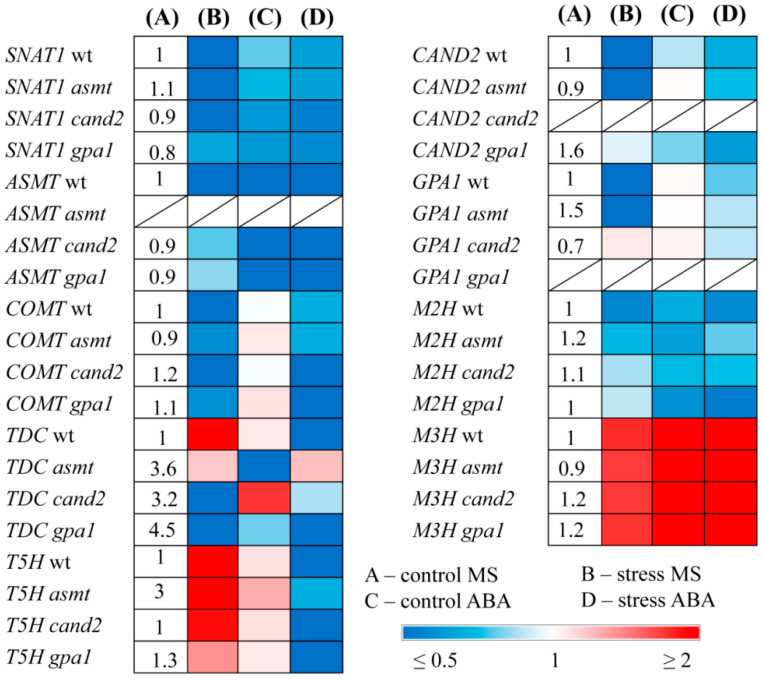
The effect of high light stress and ABA on the expression of melatonin metabolism and signaling genes in MT mutants. WT and mutant plants were grown on a Murashige and Skoog (MS) medium in Petri dishes for two weeks under a 16 h light/8 h dark photoperiod at 23 °C with 60 μmol m^−2^ s^−1^ (control). ABA was used for treatment at a concentration of 50 μM. Experimental plants were exposed to high light (HL) for 24 h at 600 μmol m^−2^ s^−1^ (stress). The numbers in the “A” column indicate the baseline ratio of the expression of each gene in the wild type and mutant without treatments.

To elucidate the regulatory interactions between ABA and melatonin, we next examined the responses of MT-related genes to ABA treatment in WT and MT mutants (Figure 2). Under mild light, exogenous ABA drastically down-regulated *ASMT* (4 fold), and to a lesser extent, *SNAT1* both in WT and MT mutants and did not change the transcript accumulation of *COMT* and *T5H*. TDC expression varied: it was not significantly affected in WT and *gpa1* but was reduced in *ASMT* and slightly increased in *cand2*. It should be noted, however, that the steady state levels of *TDC* transcripts in untreated MT mutants were three to four-fold higher than in WTs.

Under excessive irradiation, ABA maintained the expression of *SNAT1* and *COMT* at a higher level as compared to untreated stressed plants, probably due to the stress mitigating effect of ABA. *T5H* was strongly inhibited in the WT and all MT mutants except *ASMT,* despite an elevated steady state transcript level in this mutant. *TDC* transcript levels were decreased in the WT and *gpa1* and did not substantially differ from the control levels in *ASMT* and *cand2.* However, *ASMT* expression was still downregulated by the hormone in WT and MT mutants under either control or stress conditions. These results indicate that ABA constantly suppresses *ASMT* in the context of differential responses of other MT biosynthesis genes.

The interactions between ABA and melatonin may also be mediated by the expression of MT catabolism genes, given that MT metabolites may have specific functions in plants. Melatonin is converted to 2-hydroxymelatonin (2-OM) and cyclic 3-hydroxymelatonin (3-OM) by members of the 2-oxoglutarate-dependent enzyme family M2H and M3H [29]. The expression of *M2H*, encoding melatonin 2-hydroxylase (M2H), was slightly reduced in the WT and, to a lesser extent, in MT mutants upon exposure to HL or ABA and upon combined treatment with ABA and HL. In contrast, a second gene of plant melatonin metabolism, encoding melatonin 3-hydroxylase (*M3H*), was upregulated 6- to 10-fold under HL. Moreover, its transcripts increased 20- to 40-fold in WT and MT mutants after ABA treatment under mild and high irradiation. These data support the idea that ABA may antagonistically regulate MT levels, modulating the expression of MT catabolism genes.

We also tested whether the putative MT signaling genes *CAND2* and *GPA1* responded to ABA treatment. Exogenous ABA did not affect *CAND2* and *GPA1* expression under control radiation and contributed to their slightly reduced levels in high light conditions. 

### 2.3. ABA Mutations Affect Melatonin Content and the Expression of MT-Related Genes

To further delineate possible links between melatonin and ABA, we assessed the effect of modulated endogenous ABA status on the responses of MT biosynthesis and signaling genes to stress and MT treatment This study included ABA biosynthesis mutants *aba2* and *aba3* with the lowered level of endogenous ABA [30] and mutants with impaired *ABI3, ABI4,* and *ABI5* genes encoding three well-characterized transcription factors. These regulators of ABA signaling are members of the B3-, APETALA2-(AP2), and basic leucine zipper-(bZIP) domain families, respectively, which were shown to regulate overlapping subsets of ABA-inducible genes [31].

**Figure 3:**
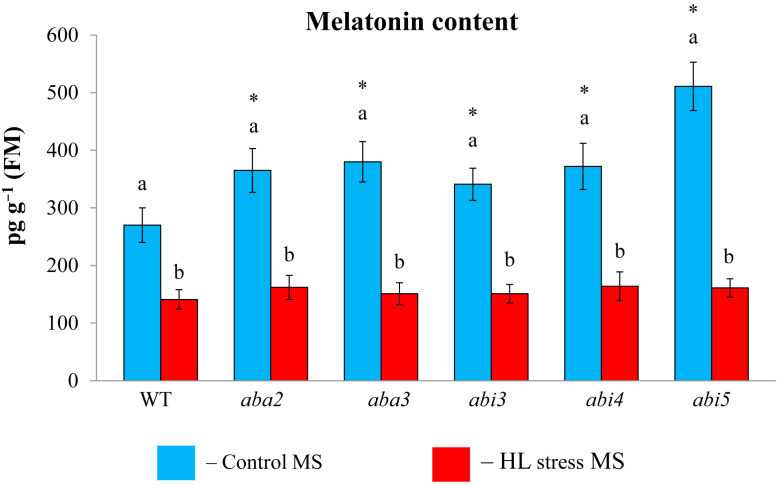
The effect of high light stress on melatonin content. WT and mutant plants were grown on a Murashige and Skoog (MS) medium in Petri dishes for two weeks under a 16 h light/8 h dark photoperiod at 23 °C with 60 μmol m^−2^ s^−1^ (control). Experimental plants were exposed to high light (HL) for 24 h at 600 μmol m^−2^ s^−1^ (stress). The data presented in the figure are the mean values (*n* ≥ 3). Error bars represent SEs. Different letters denote statistically significant differences among variants within the same genotype at *p* < 0.05, and asterisks indicate statistically significant differences between the mutants and the wild type under corresponding type of treatment at *p* < 0.05 (*t*-test).

Considering that the effect of MT treatment may depend on the level of endogenous MT, we first assessed melatonin content in ABA mutants under control conditions and under photooxidative stress (Figure 3). All the mutants displayed elevated steady state MT levels (2.5 fold) as compared to the WT, especially in *abi5,* consistent with the increased steady state levels of *ASMT* transcripts in this mutant. Following HL exposure, melatonin content decreased in the mutants with altered ABA status.

**Figure 4:**
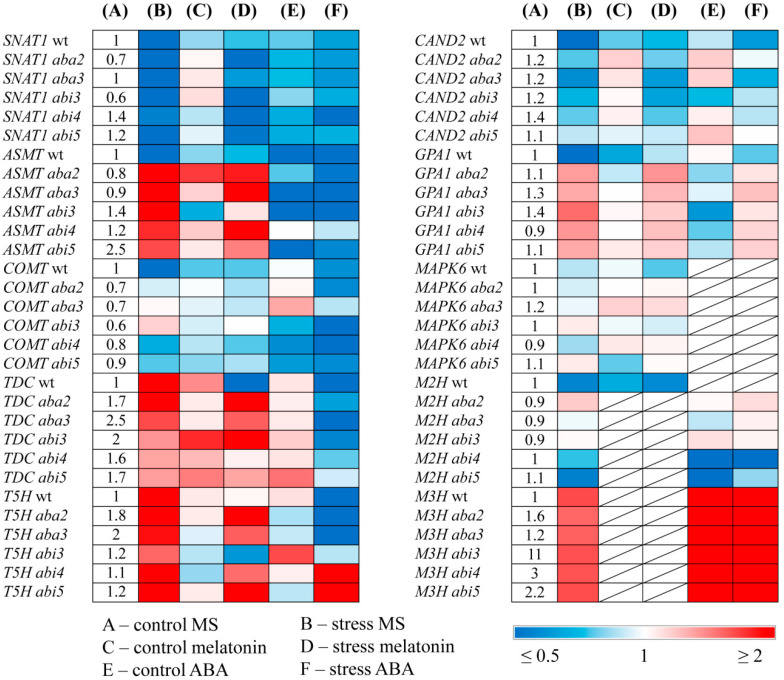
The effect of high light stress, melatonin, and ABA on the expression of melatonin metabolism and signaling genes in ABA mutants. WT and mutant plants were grown on a Murashige and Skoog (MS) medium in Petri dishes for two weeks under a 16 h light/8 h dark photoperiod at 23 °C with 60 μmol m^−2^ s^−1^ (control). Melatonin and ABA were used for treatment at a concentration of 50 μM. Experimental plants were exposed to high light (HL) for 24 h at 600 μmol m^−2^ s^−1^ (stress). The numbers in the “A” column indicate the baseline ratio of the expression of each gene in the wild type and mutant without treatments.

The expression of MT synthesis genes *SNAT* and *COMT* was downregulated under HL in the mutants, though less than in the WT or did not change (Figure 4). However, in contrast to the WT, *ASMT* transcript levels increased, especially in *aba3* and *aba2* (five and six-fold, respectively) and remained elevated after combined treatment with HL and melatonin. Thus, the defects of ABA synthesis or signaling contributed to the induction of *ASMT.* These results imply that *ASMT* expression negatively correlated with ABA content and signaling. *T5H* and *TDC* were also activated by stress and stress+MT, in ABA mutants, although, in WT, the stress-induced elevation of both genes was mitigated by MT.

HL mildly reduced the expression of *CAND2* and did not reliably affect *GPA1* in ABA mutants, in contrast to the WT, in which both genes were dramatically downregulated (three to four-fold). Under combined MT+HL treatment, no further changes in the *CAND2* and *GPA1* expression occurred in tested ABA mutants, while, in WT plants, the transcript levels of both genes increased as compared to those in untreated stressed plants. Hence, the expression patterns of *CAND2* and *GPA1* under stress conditions depend on the ABA status with ABA deficiency promoting the increased expression of MT signaling genes. The results also suggest the possible involvement of additional factors in ABA-dependent regulation of the *GPA1* expression since the expression pattern of *GPA1* in ABA mutants did not strictly follow that of *CAND2.*

Given that the melatonin signaling pathway may act through the mitogen-activated protein kinase (MAPK) cascade, [6] we also analyzed expression profiles of *MAPK6.* However, no statistically significant changes occurred following HL or HL+MT exposure in either WT or ABA mutants.

### 2.4. MT Treatment Alters the Expression of ABA-Related Genes in MT Mutants 

**Figure 5:**
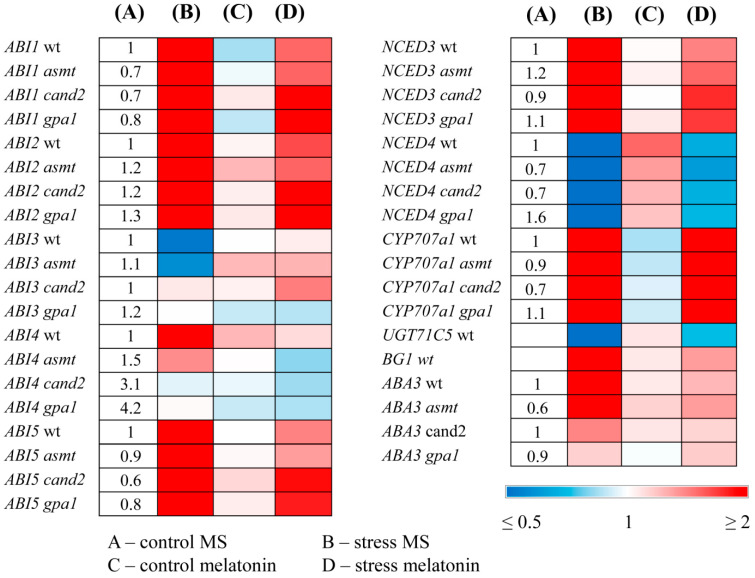
The effect of high light stress and melatonin on the expression of ABA metabolism and signaling genes in MT mutants. WT and mutant plants were grown on a Murashige and Skoog (MS) medium in Petri dishes for two weeks under a 16 h light/8 h dark photoperiod at 23 °C with 60 μmol m^−2^ s^−1^ (control). Melatonin was used for treatment at a concentration of 50 μM. Experimental plants were exposed to high light (HL) for 24 h at 600 μmol m^−2^ s^−1^ (stress). The numbers in the “A” column indicate the baseline ratio of the expression of each gene in the wild type and mutant without treatments.

Next, we studied whether melatonin regulated the expression of ABA-related genes in MT mutants. In wild type plants, key ABA synthesis and signaling genes *NCED3, ABA3, ABI1, ABI2, ABI4,* and *ABI5* were upregulated, and *ABI3* was downregulated by excess irradiation, whereas melatonin attenuated these responses (Figure 5). Such alleviation may indicate a more stable status of the plant exposed to melatonin, when the stress-induced response of ABA genes is less pronounced. At the same time, this reaction may be the result of competitive relations between these regulators for a common set of transcription targets under stress, leading to the partial repression of ABA genes by melatonin. Of note, *NCED4*, which plays a major role in the degradation of beta-carotene, a substrate for ABA synthesis [32], was strongly downregulated by HL stress in accord with the stress protective role of ABA and, to a lesser extent, following MT+HL treatment alleviating the effects of stress.

The catabolism of ABA from ABA to phaseic acid is catalyzed by a cytochrome P450 monooxygenase (P450) encoded by *CYP707As* [33]. ABA can also be glucosylated by a UDP-glucosyltransferase encoded by *UGT71C5* to ABA-glucose ester (ABA-GE), which is an inactive form of ABA [34]. ABA-GE, in its turn, can rapidly be transformed to active ABA via *AtBG1* and *AtBG2* encoded β-glucosidases when the environment conditions change. MT treatment under HL did not substantially affect *CYP707A* transcript levels compared to their increased values under HL. The *UGT71C5* expression was significantly downregulated, while that of *AtBG1* dramatically upregulated under HL, which may contribute to increased ABA levels, and these alterations were attenuated by MT. Therefore, melatonin treatment primarily affects genes involved in the metabolism of reversible forms of ABA.

In the melatonin-deficient *ASMT* mutant, the response of ABA genes to stress and melatonin was similar to that of the wild type, although less pronounced for some genes. However, in *cand2* and *gpa1* mutants, *ABI4* was not regulated by either stress or melatonin. Moreover, MT insensitive *cand2* and *gpa1* exhibited elevated steady state levels of *ABI4* transcripts (three and four-fold, respectively, as compared to the WT), suggesting that *ABI4* is constitutively derepressed in the *cand2* and *gpa1* background, and further response to stress treatment is not possible. Steady state levels of *ABI4* transcripts in MT deficient *ASMT* were also significantly increased (1.5-fold). These results imply that the transcription factor ABI4 may play a role in the interactions between ABA and melatonin.

### 2.5. ASMT as a Potential Target Gene for ABI4 Under HL Stress 

Finally, we tested whether MT metabolism and signaling genes were transcriptionally altered in ABA mutants (Figure 4). Steady state levels of *TDC* and *T5H* transcripts were significantly elevated, and that of *COMT, SNAT,* and *GPA1* were reduced in *aba2* and *aba3* mutants relative to the WT. *ASMT* and *TDC* transcript abundance was increased in *abi5* and *abi3*, while the expression of *CAND2* was not significantly altered.

Under the ABA treatment or ABA treatment coupled with HL, the expression of *SNAT* and *COMT* slightly decreased in WT and ABA mutants as compared to untreated samples. The ABA+HL application also down regulated *TDC* and *T5H* in WT, *aba2*, *aba3* and abi3 as compared to their stress-induced levels but had no impact in *abi4* and *abi5*. These data suggest that ABA may act through ABI4 and ABI5 transcription factors in the stress-dependent regulation of *TDC* and *T5H*. It should be noted, however, that *TDC* and *T5H* have also been identified as participants in IAA biosynthesis. Therefore, the ABA-dependent expression of these genes may be related to ABA/auxin interactions, given that the expression of downstream *SNAT* and *COMT* in the melatonin biosynthetic pathway followed that of the WT in all ABA mutants tested upon ABA+HL.

ABA also negatively regulated *ASMT* transcript accumulation, under both mild and high light in WT plants and in *aba2, aba3, abi3,* and *abi5*. However, *ASMT* did not respond to ABA treatment in the *abi4* mutant. These results suggest that *ASMT* can be a potential target gene for ABI4, thus supporting the notion that the transcription factor ABI4 is a key player in the interactions between ABA and melatonin.

Furthermore, the *ABI4*-disrupted mutant showed a two to three-fold decrease in *M2H* transcript accumulation following ABA treatment and a two-fold increase in transcript levels compared to the dramatically ABA-induced *M3H* expression in the WT and other ABA mutants. These results confirm that ABI4 is an important factor in the regulation of the interactions between melatonin and ABA. However, the exact biological meaning of ABA-dependent shifts in the expression of melatonin catabolism genes remains to be clarified, taking into account that, on the one hand, 2-OHM triggers the production of ROS [35], and, on the other hand, that 3-OHM is a powerful antioxidant exhibiting even higher antioxidant activity than melatonin [5].

### 2.6. In Silico Promotor Analysis of ASMT Gene

A role for the ABI4 transcription factor in the regulation of MT synthesis implies that promoter regions of MT-related genes contain *cis*-regulatory elements that are potential binding sites for ABI4. ABI4 was shown to bind a number of DNA sequences, including Coupling Element 1 (CE1) CACCG [36], two ABE motifs GC(C/G)GCTT(T) [37], B ELEMENT CGTGAT [38], and S-box and S-box similar sequence CACYKSCA [39]. A motif CCAC has also been suggested to bind ABI4 particularly when adjacent to, or overlapping with, a G box motif [40]. In addition, DRE and ABRE core sequences have the potential to be bound by ABI4 [37].

**Figure 6:**
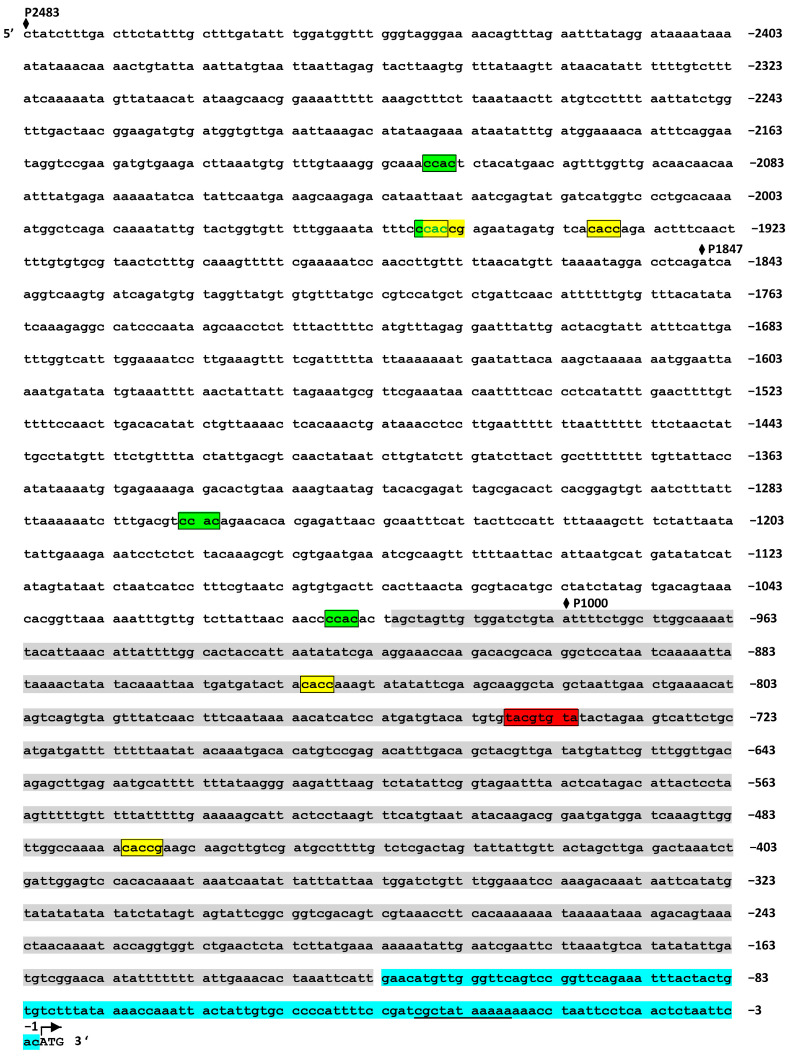
The promoter region of the *ASMT* gene, 2483 bp in length, including the 5′-UTR region. The nucleotide sequence for the 5′-UTR is highlighted in blue. The basal promoter sequence of the gene, −1000 bp from the ATG initiation codon, identified using the PlantProm DB program, is shown in gray. CCAC motifs are shown in green. ABI4-binding elements CE1 CACCG are shown in yellow. ABRE-like motif TACGTGT is shown in red. According to the program https://agris-knowledgebase.org/ (accessed on 2 October 2024), the length of the promoter of this gene can reach a longer region of 2483 bp. The TATA box sequence is underlined, and the ATG start codon is indicated by arrow. The start of 5′-deletion fragments P1000, P1847, and P2483 are marked by black rhombus.

A detailed in silico analysis of the *ASMT* promoter region within 1000 bp upstream from the ATG start codon (basal part) showed one potential ABI4-binding element CE1 (nt −467 to −472) (Figure 6). The binding affinity of CE1 (CACCG) is considered higher than that observed with other DNA-binding sites (Finkelstein et al., 2011). We also identified one ABRE-like element (nt −742 to −749) within the basal part. Five more elements, one CE1 (nt 1953 to 1958) and four CCAC motifs (nt −1005 to −1010; −1261 to −1266; −1954 to −1959; and −2113 to −2118) were found in the distal region of the *ASMT* promoter as its maximum length can reach 2483 bp according to the AGRIS database (https://agris-knowledgebase.org/AtcisDB/getpromseq.html?id=At4g35160 (accessed on 2 October 2024)). We therefore hypothesize that *ASMT* could be a direct transcriptional target of ABI4.

It should be noted that the sequence recognition by ABI4 may be more flexible than known canonical sequences. According to the results of [31], many of the direct ABI4 transcriptional targets do not contain the previously characterized ABI4 binding motifs. Nevertheless, some of them were able to interact with ABI4 in electrophoretic mobility shift assays. Moreover, ABI4-regulated promoters could be synergistically induced by specific combinations of ABI4 and some bZIP factors, even when no canonical ABI4 binding sites were present. Given a relatively small number of direct transcriptional targets for ABI4 within the proximal part, we can also hypothesize that the *ASMT* promoter can be regulated indirectly via *cis*-regulatory elements recognized by ABI4-inducible transcription factors.

A functional characterization of the *ASMT* promoter was carried out by testing the ability to drive the expression of the reporter β-glucuronidase (*GUS*) gene. To this end, three 5′-deletion fragments (−1000 bp, −1847 bp, and −2483 bp) upstream of the *ASMT* translation start codon were fused to the open reading frame of the *GUS* reporter gene. ABA treatment (50 µM of ABA for 6 h) of *ASMT pro*: *GUS* fusions in the wild type background resulted in a considerable decrease in the accumulation of *ASMT pro*: *GUS* transcripts as compared to untreated leaf disks (Figure S2). The signal also decreased in plants containing 1000 bp of the *ASMT* promoter fragment in the *abi4* background. At the same time, plants containing the 1847 bp and 2483 bp fragments in the *abi4* background did not differ from untreated plants when exposed to ABA, suggesting that ABI4 was required for ABA-induced decrease in *ASMT* promoter activity. Given that the distal region of the *ASMT* promoter includes one CE1 and four CCAC motifs, it was concluded that ABI4 can directly play a role in the ability of the ASMT promoter to respond to ABA treatment.

## 3. Discussion

Overall, we found that melatonin and ABA are able to mutually influence the expression of the genes involved in their metabolism and signaling. In particular, the *ASMT*, which encodes enzyme of the final step of melatonin biosynthesis, was suppressed by melatonin treatment under both mild and HL in the wild type. However, the expression remained virtually unchanged in the mutants with the disrupted genes for ABA synthesis and signaling under mild light and was even upregulated under combined MT+HL treatment. Therefore, deficiencies in ABA content or perception contribute to the increased expression of *ASMT* gene, which has been proposed to be stress regulated [41].

In parallel, the *ASMT* was downregulated by ABA treatment in the wild type plants and to a lesser extent in *aba2, aba3, abi3,* and *abi5* mutants with the compromised ABA status. But the *ASMT* did not respond to ABA in *abi4* with the inactivated gene for transcription factor ABI4. We therefore hypothesize that the application of ABA induces repression in *ASMT* promoter activity in the WT via interaction with the ABI4 transcriptional factor, and this repression is absent in the *abi4* mutant. These results are consistent with the notion that *ASMT* is a potential target gene for ABI4, which is probably involved in melatonin biosynthesis as a negative regulator.

It should be noted that ABI4 has been suggested to act as both a transcription activator and a repressor, which is determined by its interaction with other proteins, protein modification, or binding to discrete DNA binding sites leading to either the activation or repression of target genes [37]. ABI4 binds to a number of GC-enriched DNA sequences, providing the regulation of target genes involved in a large functional spectrum of activities. At least two Coupling Element1 (CE1) sequences (CACCG) are present in the *ASMT* promotor region and one ABRE-like element (TACGTGTA), in addition to seven CCAC motifs, which also correlated with *ABI4* signaling [40].

Furthermore, in this study, we found that *ASMT pro*: *GUS* fusions did not respond to ABA treatment in the *abi4* mutant background, although ABA decreased *ASMT* promoter activity in WT background. These results imply that a functional ABI4 is necessary for the ABA-dependent regulation of *ASMT* expression. Consistent with the fact that ABI4 is involved in ABA–melatonin crosstalk, MT-insensitive *cand2* and *gpa1* exhibited increased steady state levels of *ABI4* transcripts, suggesting that *ABI4* is constitutively derepressed in genotypes defective in MT signaling.

ABI4 has long been known as a versatile factor that functions in diverse pathways and is tightly regulated at the transcriptional and post-transcriptional levels. The expression of *ABI4* was shown to be redundantly regulated by ABRE (ABA-responsive element)-binding transcription factors AREB1/ABF2, AREB2/ABF4, and ABF3 [42], which, in their turn, promote ABA-mediated chlorophyll degradation by directly binding to the promoters of chlorophyll catabolic genes such as *NYE1*/*SGR1*, *PAO,* and *NYC1* [43]. Considering that Abi4 showed substantial synergy when combined with ABI5 or other ABF transcriptional factors [31], it is possible that the overexpression of *ABI4* in *cand2* and *gpa1* mutants may induce accelerated chlorophyll degradation following ABA treatment under mild light. Concomitantly, ABA contributed to its partial retention under stress conditions. Although, the absolute values of chlorophyll content remained reduced in the mutants as compared to WT plant levels. Further studies are required to accurately clarify such an unusual effect of ABA on the chlorophyll content.

Although MT may act directly as an anti-stress agent, the complexity of the MT-dependent gene expression suggests that MT mediates many important functions through a melatonin receptor(s) and downstream signal transduction pathways in plants. To date, studies of the melatonin- CAND2-GPA1-signaling pathway have documented its involvement in osmotic, salt, drought and HL stress tolerance, in addition to biotic stresses. Here, we showed that an intricate network of ABA-related genes under HL, at least in part, depends on CAND2. This finding is particularly relevant for ABA signaling, since, in contrast to WT, the expression levels of ABA signaling genes in *cand2* and *gpa1* mutants did not respond significantly to MT treatment.

The identification of molecular links between MT and ABA synthesis and signaling provides a basis for understanding how disruptions of these two regulators can feed back to influence each other. According to the results of this study, inactivating components of the MT signaling circuit results in compensatory effects in ABA signaling. On the other hand, deficiencies in ABA synthesis and signaling entail the increased expression of MT synthesis genes and elevated steady state MT levels. The existence of such compensatory mechanisms gives rise to a certain parallelism in the action of these two regulatory molecules and can have a broad impact beyond the immediate activity of the inactivated components.

In this context, ABA-dependent modulations in the expression of melatonin catabolism are of special interest. ABA treatment promoted the downregulation of *M2M*, which may result in decreased levels of 2-OM with its ROS-inducing mode of action and consequently decreased ROS production. In parallel, *M3H* upregulation, particularly in the *abi4* mutant, may trigger 3-OM overproduction and enhance antioxidant activity. Therefore, the ABA-induced decrease in MT content, which can be regarded as a manifestation of the antagonistic relationship between ABA and MT, may actually lead to an increase in antioxidant capacity and stress resistance.

The functional redundancy in the effects of MT and ABA allows the plant to trigger alternative pathways in response to environmental challenges or developmental demands. At the same time, they provoke antagonism between ABA and MT under both control and stress conditions. As we have already outlined, examples of antagonistic relations between MT and ABA were shown for a number of plant species [11,12,13,14,15,16,17]. On the other hand, synergism in MT and ABA effects was also demonstrated in several studies [18,19,20,21,22]. Moreover, the dual role of melatonin as a signaling molecule in ABA–melatonin interplay was revealed during the seed formation and seed germination of *Arabidopsis*. Knock-out mutants for melatonin receptor *PMTR1* contained higher ABA concentrations in developing seeds but accumulated lower ABA levels in dry and imbibed seeds than the wild type Col-0 [44]. Hence, the multiplicity of possible functions defined by ABA–melatonin crosstalk imply multivariant responses depending on the physiological state and genetic background.

The interactions between melatonin and ABA are likely to be integrated with other hormone signals. The mutual impact of ABA, gibberellic acid (GA), and MT has been shown for cucumber. Exogenous melatonin increased the expression of ABA catabolism genes (monooxygenase, CYP707A1, and CYP707A2) and GA biosynthesis genes (GA20ox and GA3ox) and also suppressed the ABA biosynthesis gene 9-cis-epoxycarotenoid dioxygenase (NCED2). This change led to a decrease in ABA levels and an increase in GA_3_ and GA_4_ content and accelerated seed germination [15]. A similar picture was observed during the germination of cotton seeds (*Gossypium hirsutum* L.) under salt stress conditions [45].

In ryegrass (*Lolium perenne* L.), exogenous melatonin reduced the ABA content by suppressing the expression of biosynthesis (*LpZEP* and *LpNCED1*), downregulated ABA signaling genes (*LpABI3* and *LpABI5*), but activated cytokinin (CK) synthesis genes (*LpIPT2* and *LpOG1*), thereby increasing the content of endogenous CK and activating CK signaling circuits [46]. In our prior research, we showed that melatonin may act synergistically with CK in *Arabidopsis* via the melatonin-mediated activation or repression of CK synthesis and signaling genes [16]. Conversely, CK was involved in expression regulation of the genes for melatonin metabolism and signal transduction, with the melatonin biosynthesis gene *ASMT* being a key component in the crosstalk between the CK and MT metabolic pathways. In this study, we revealed that *ASMT* is a potential target for ABI4 in ABA–melatonin intersection. Therefore, the fluctuation in melatonin activity induced by various hormones may, at least in part, be mediated by ASMT. These data are consistent with RNA sequencing and qRT-PCR data obtained by [47], which showed that, in the walnut, *Juglans regia*, members of the *ASMT* gene family participate in regulatory networks of phytohormones such as ABA, salicylic acid, and CK, regulating flower development and stress tolerance. In addition, the in silico analysis of the promoter regions revealed 11 hormone-response motifs, including the ABA response element ABRE, the auxin response element AuxRR-core, jasmonic acid (JA) response elements CGTCA and TGACG, gibberellin response element P-box, and salicylic acid response element TCA.

Hormone-like behavior of MT while interacting with classical hormones, as well as the participation of the PMTR1/CAND2 receptor in such reactions, makes it possible to classify melatonin as a new phytohormone, especially when it acts in physiologically effective concentrations using high-affinity binding sites. At the same time, MT is believed to emerge as the first-line antioxidant capable of operating in cardinally higher concentrations [48,49]. The evolution in modes of action and the acquisition of secondary hormone-like properties have required the incorporation of melatonin into a highly interconnected web involved in hormone metabolism and signaling [50]. This process contributed to the generation of joint pathways that ensured the inclusion of melatonin in the plant hormone system. The interplay between ABA and melatonin, resulting from a considerable overlap in effects, is an example of such integration, with the MT-dependent regulation of ABA genes via CAND2/GPA1 signaling circuit and ABI4/ASMT acting as elements of this bond.

Further unraveling the molecular network that links specific melatonin affects with hormonal pathways will undoubtedly improve our understanding of melatonin function in plants. It will be of great interest to investigate global changes in the expression of multiple elements of hormonal synthesis, degradation, and signaling regulated by melatonin with the special emphasis on identification potential points of crosstalk between melatonin and phytohormones. Basic knowledge of transcriptional changes, which are common outcomes of phytohormone–melatonin crosstalk, may finally be translated into novel strategies for manipulating plant growth and defense.

## 4. Materials and Methods

### 4.1. Plant Materials and Stress Treatments

*Arabidopsis thaliana* WT (ecotype Columbia) and mutant lines *aba2-1* (NASC 156), *aba3-1* (NASC 157), *abi3* (NASC 503216)*, abi4* (NASC580095), *abi5* (NASC 513163)*, asmt* (NASC 680911), *cand2* (NASC 678658), and *gpa1* (NASC 6534) were used in this study. Seeds were surface-sterilized and stratified for 48 h at 4 °C in the dark. Plants were grown on a half-strength Murashige and Skoog (MS) medium with 1% sucrose and 0.5% agar in a growth chamber at 23 °C with a 16 h photoperiod and photosynthetic photon flux density of 60 μmol m^−2^ s^−1^. Two-week-old plants were pretreated with 50 μM of melatonin for 72 h or 50 μM of ABA for 24 h. Prolonged pre-treatment was chosen because of the amphiphilic nature of melatonin, which implies relatively low penetrating ability. For HL treatment, plants were exposed for 24 h under the HPI-T2 2000 W/646 lamp (Philips, Netherlands) with a luminous energy flux of 600 μmol m^−2^ s^−1^. A combination of air and water cooling was used to minimize the thermal impact of the lamp. Control plants were left under growing conditions. At the end of the exposure, plants were immediately frozen in liquid nitrogen and subjected to further analysis.

*Arabidopsis thaliana* WT (ecotype Columbia) and mutant lines *aba2-1* (NASC 156), *aba3-1* (NASC 157), *abi3* (NASC 503216)*, abi4* (NASC 580095), *abi5* (NASC 513163)*, asmt* (NASC 680911), *cand2* (NASC 678658), and *gpa1* (NASC 6534) were used in this study. Seeds were surface-sterilized by sodium hypochlorite (5%) and stratified for 48 h at 4 °C in the dark. Plants were grown on a half strength Murashige and Skoog (MS) medium with 1% sucrose and 0.5% agar in a growth chamber at 23 °C with a 16 h photoperiod and photosynthetic photon flux density of 60 μmol m^−2^ s^−1^. Two-week-old plants were transferred to paper filters with a liquid MS medium and were pretreated with 50 μM of melatonin for 72 h or 50 μM of ABA for 24 h. Prolonged pre-treatment was chosen because of the amphiphilic nature of melatonin, which implies relatively low penetrating ability. For HL treatment, plants were exposed for 24 h under the HPI-T2 2000 W/646 lamp (Philips, Amsterdam, The Netherlands) with a luminous energy flux of 600 μmol m^−2^ s^−1^. A combination of air flow and water flow cooling was used to minimize the thermal impact of the lamp. This action allowed the temperature to be maintained at 23 °C at both leaf and root levels. Control plants were left under growing conditions. At the end of the exposure, plants were immediately frozen in liquid nitrogen and stored at −80 °C for further analysis. 

### 4.2. RNA Extraction and Qrt-PCR

Total RNA was extracted from frozen samples utilizing the TRIzol (Thermo Fisher Scientific, Waltham, MA, USA) method. cDNAs synthesis was performed from 2 μg of total RNA using a mixture of oligo (dT) primers and random hexamers. Relative gene expression levels were quantified via qRT-PCR using LightCycler 96 (Roche, Rotkreuz, Switzerland) with the hot start SYBR Green I technology. A standard thermal profile for all PCRs included the following steps: 95 °C for 5 min, 40 cycles of 95 °C for 15 s, 58 °C for 15 s, and 72 °C for 25 s. The nuclear-encoded polyubiquitin *UBQ10* gene was used as an internal control. Primers used for qRT-PCR are presented in Table S1.

### 4.3. Melatonin Measurement

The determination of endogenous melatonin concentration was performed as described by Lee and Back [51] utilizing an ELISA Kit CEA908GE (Cloud-Clone Corp., Katy, TX, USA) according to the manufacturer’s protocol. The optical density was measured at 450 nm with a Multiskan MS Microplate Reader LabSystems 352 (Thermo/LabSystems, Pittsburg, PA, USA).

### 4.4. Quantifications of Chlorophyll Content

Pigments were extracted from plant rosette leaves using 80% (*v*/*v*) acetone. The concentrations of chlorophyll (A + B) and carotenoids were determined by examining the absorbance of 440, 649, and 665 nm in the centrifugated supernatant as described by Lichtenthaler [52].

### 4.5. Determination of TBARs

Secondary products of membrane lipid peroxidation (TBARs) were determined by the reaction with thiobarbituric acid, as described by Heath and Packer [53]. The measurement was performed on a spectrophotometer (Pharmacia Biotech ultrospec 2000, London, UK) at wavelengths of 532 and 600 nm. The concentration of TBARs (μmol/g of fresh weight) was calculated using the formula: C = D/EL, where C is the concentration of MDA, μmol, D is the optical density, and E is the molar extinction coefficient equal to 1.56 × 105 cm^−1^ M^−1^. The amount of TBARs was calculated in μmol/g of wet weight.

### 4.6. In Silico Promoter Analysis

The genomic sequence of *ASMT* gene upstream of the ATG codon was obtained from *arabidopsis.org* database. The *ASMT* promoter and *cis*-acting elements were predicted using the PlantProm DB (accessed on 1 October 2024) [54] database and the Arabidopsis Gene Regulatory Information Server (AGRIS) (accessed on 1 October 2024) [55] databases, respectively. The nucleotide sequence analysis and the localization of regulatory elements were performed with Vector NTI Advance 9.0 (Invitrogen, Waltham, MA USA) and visualized in Figure 6.

### 4.7. Construction of the ASMT Pro: GUS Reporter Gene Fusions and the Generation of ASMT Pro: GUS Transgenic Plants 

The generation of *ASMT pro: GUS* transgenic plants were performed as we previously described [56]. In brief, three fragments (1000 bp, 1847 bp, and 2483 bp) upstream of the translation start codon of *ASMT* were amplified from the genomic DNA via a polymerase chain reaction using different forward (P1000: 5’-aatggatccAGCTAGTTGTGGATCTGTAA-3’; P1847: 5’-aatggatccATCAAGGTCAAGTGATCAGAT-3’; P2483: 5’-atggatccCTATCTTTGACTTCTATTTGCT-3’) and general reverse (5’-aatctgcagGGTGAATTAGAGTTGAGGAAT-3’) primers carrying *Bam*HI and *Pst*I restriction sites (underlined), respectively. The resulting *Bam*HI/*Pst*I fragments were cloned upstream of the promoterless *uidA* reporter gene (*GUS*) of the binary vector pCambia-1381Z (Clontech, San Jose, CA, USA), producing pCambia *ASMT pro*: *GUS* constructs. The constructs obtained were introduced into *Agrobacterium tumefaciens* strain GV3101 (C58) (GoldBio, St Louis, MO, USA) using the freeze-thaw method. Five-week-old wild type *Arabidopsis* plants in the Col-0 and *abi4* background were transfected with *A. tumefaciens* cells harboring P1000, P1847, and P2483 plasmids by the floral dip method. T1 transgenic seeds from each transformant plant were tested for germination on a half MS medium containing 0.8% (*w*/*v*) phyto agar (Duchefa Biochemie, Haarlem, The Netherlands) and 30 mg/L of hygromycin (Hyg). The Hyg-resistant seedlings were then grown in soil for further analysis of *ASMT pro: GUS* expression.

### 4.8. Statistical Data Processing

All the experiments were repeated at least three times with similar results. Statistical analyses of gene expression data were performed with ANOVA with post hoc Holm multiple-comparison calculation using the online calculator (astatsa.com/OneWay_Anova_with_TukeyHSD/) (accessed on 1 October 2024). All data are presented as the mean values ± standard errors (SEs).

## 5. Conclusions

A plant’s ability to cope with stresses is highly correlated with two regulatory molecules, melatonin and ABA, which exhibit parallelism in their action. The overlapping effects of ABA and melatonin on the expression of their signaling and metabolic genes may provide a basis for understanding mechanisms of their interaction in plant responses to adverse environmental conditions. Here, we showed that compromised MT signaling results in compensatory effects in ABA signaling, whereas deficiencies in ABA synthesis and signaling cause the increased expression of MT synthesis genes and elevated steady state MT levels implying that ABA and melatonin act antagonistically. On the other hand, the ABA-dependent regulation of melatonin catabolism genes *M3H* and *M2H* promoting a decrease in MT levels may, in turn, modulate the content of MT metabolites and, as a result, augment plant antioxidant activity. This indirect effect highlights the multifaceted output of ABA-MT interplay.

Changes in the expression of *M2H* and *M3H* were especially pronounced in *abi4,* inferring that this transcription factor is a negative regulator of ABA-dependent changes in MT content. Furthermore, ABA induced the downregulation of the MT synthesis gene *ASMT*. However, the repression was absent in the *abi4* mutant suggesting that *ASMT* can be a potential target gene for ABI4. An intricate network of ABA and MT-related genes under HL, at least in part, is dependent on the MT receptor CAND2. *ABI4* was not regulated by either stress or melatonin in *cand2* and *gpa1,* which had elevated steady state *ABI4* transcript levels, specifying that *ABI4* is constitutively derepressed. Overall, our results suggest that the functional redundancy of the effects of ABA and MT may contribute to their antagonistic relationship, superimposed on their exclusive transcriptional programs.

## Data Availability

Data is contained within the article and Supplementary Materials.

## Supplementary Materials

The following supporting information can be downloaded at: https://www.mdpi.com/article/10.3390/ijms252212266/s1.
