# Supplementary material for: ABA and Melatonin: Players on the Same Field?
Source: Int J Mol Sci. 2024 Nov 15;25(22):12266. doi: 10.3390/ijms252212266 (PMC11594332; doi:10.3390/ijms252212266)
Supplement: Supplementary file 1 [file ijms-25-12266-s001.zip › Supplementary Materials.pdf]

Supplementary Materials

ABA and Melatonin: Players on the Same Field?

Ivan Bychkov, Natalia Kudryakova*, Elena S. Pojidaeva, Anastasia Doroshenko, Victoria Shitikova, Victor Kusnetsov

K.A. Timiryazev Institute of Plant Physiology RAS, 35 Botanicheskaya St., Moscow, 127276, Russia*

Table S1. List of primers used for RT-qPCR.

Gene name	Locus	Forward primer (5′->3′)	Reverse primer (5′->3′)
SNAT1	AT1G32070	TAC GCA ACT TGT GGA ACC ACC T	ATC ATA AAC ATC AAT CTC ACC ACC A
ASMT	AT4G35160	GCAAAGAAGCGGTCCC TCCAA	GTCCGTTCTTTGCCT GTGCTTGT
COMT	AT5G54160	GGA GTG ACG AAC ATT GCG T	TCT CGG TTC GTT CTT TGC CT
T5H	AT3G53305	GCC GTA GAA GCT GTC CTG GTA	AGT GCT GAT GGA AGA GTT GGT ATG
TDC	AT2G20340	CCT GGA GTA ACG CAT TGG CA	AGT GTT GCT GGG TTC TTG GG
CAND2	AT3G05010	ATG CGA GTG CTC AGC GAG AT	TTA TTC CCA ATC AGC GTC GAA GAA T
GPA1	AT2G26300	CGT TTG CGA GTG GTT CAG AGA T	CCA AAG CCG TCG TCC TGT AGA T
M2H	AT3G60290	AGG TTC TTT GAC TTA CCT GCC G	GTA GCA GGG AGG ATT GGA AGG
M3H	AT1G17020	CAG GAA AGA AGC CGA CAT GGA	TAG GAG GAA CGG TCG TGA TCG
ELIP2	AT4G14690	ACG GGA GAC TAG CAA TGG TT	CCT AGA AAC CAC CCG ACA CC
PSBS	AT1G44575	TTG CTT GGT GAG GCG TTG AC	GGC TCT GCT TCG TAA ATC GG
AOX1a	AT3G22370	GAT TGG AGG TAT GAG ATT CGC	CGG TGG ATT CGT TCT CTG TTT
MAPK6	AT2G43790	ATC TCC ATC AAA TCA TTC GGT CAA A	CAG TTT GCG TTC AGG AGG AGA TTA C
ABI1	AT4G26080	GAA GAA GCG TGT GAG ATG GCA	TCC CTT CCT TTC TCC GCT CA
ABI2	AT5G57050	TGG TGT TCT CGC AAT GTC AAG	CAA ATC GCA CAC TTC TTC GT
ABI3	AT3G24650	TGC CTG CTC CAA ACT ATC CG	TTA TAC TGC GAG GTT GGC GG
ABI4	AT2G40220	CAA GGA GGA AGT GGG TGT AAT AAT AA	ATC CAG ACC CAT AGA ACA TAC CG
ABI5	AT2G36270	TGG AGA GGA AGA GGA AGC AA	GTT TGA AGT CAA GGG CAC AA

NCED4	AT4G19170	CAC CGA AAC TCC GAC CAG AAA	CGG AAG GAC GTG AAG GTG GAT
CYP707a1	AT4G19230	CGT GTC CTG GAA ATG AAT TAG CC	GCA CAA TGG GCA GTC CGT TT
ABA3	AT1G16540	ACT GAG ACC GTG CTA CAA GGC T	ATC AAT CAA GAC CAT CCA CCG
UBQ10	At4G05320	GCG TCT TCG TGG TGG TTT CTA A	GAA AGA GAT AAC AGG AAC GGA AAC A

Sequence data from this article can be found in the National Center for Biotechnology Information https://www.ncbi.nlm.nih.gov/

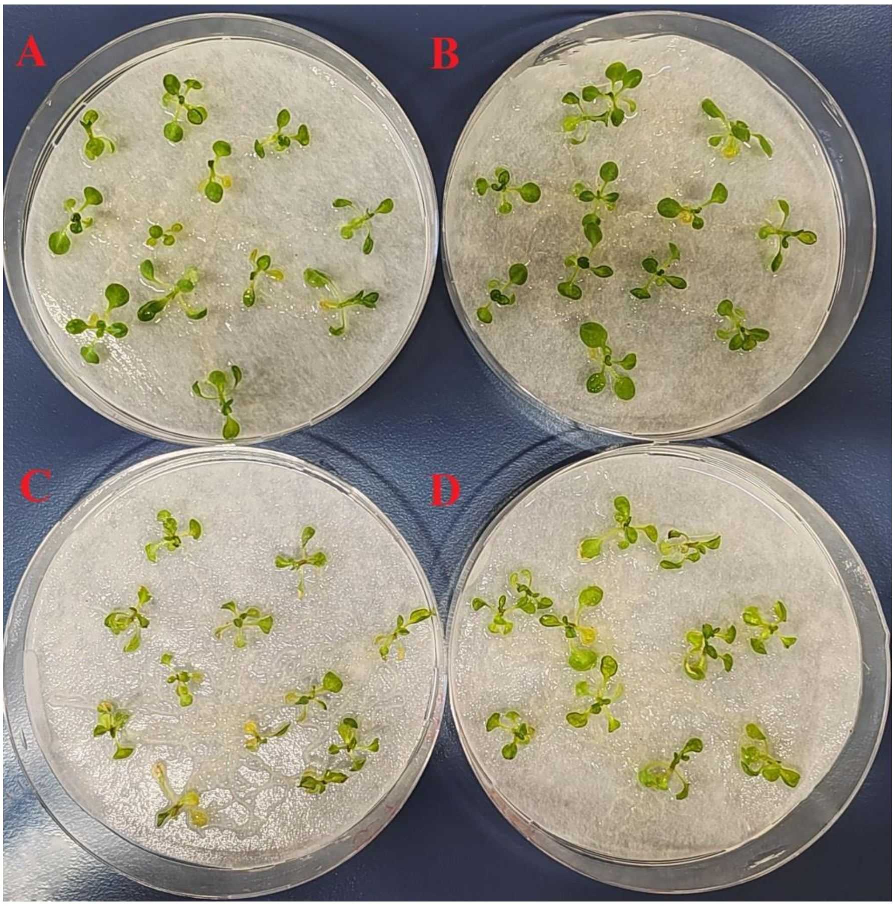

Figure S1. *Arabidopsis thaliana* plants under the action of photostress and melatonin. A – control, B – control + melatonin, C – stress, D – stress + melatonin.

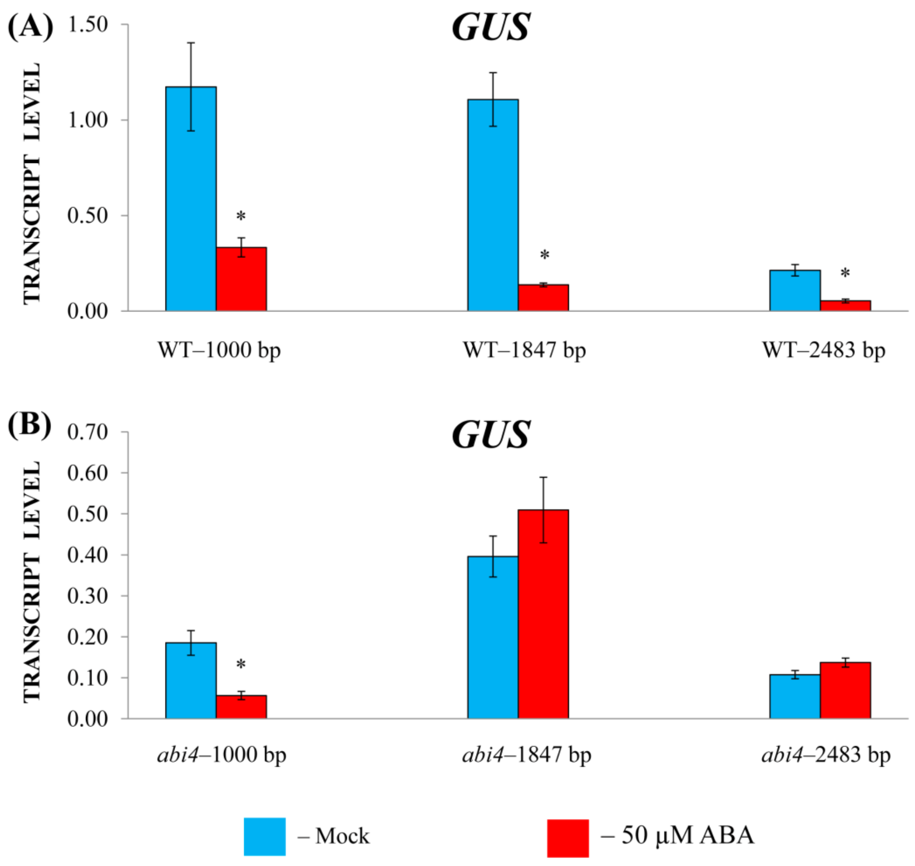

Figure S2. Expression of the reporter β-glucuronidase (*GUS*) gene in five-week-old leaves *Arabidopsis thaliana* exposed for 6 h to mock solution (A) or 50 µM ABA (B).
